# Molecular characterization and infectious clone construction of beet capulavirus 1 infecting tomato in Germany

**DOI:** 10.1099/jgv.0.002347

**Published:** 2026-10-08

**Authors:** Omid Eini, Paolo Margaria, Dennis Knierim, Claudia Pommerenke, Monika Heupel, Björn Krenz, Mark Varrelmann, Wulf Menzel

**Affiliations:** 1 Department of Phytopathology, Institute of Sugar Beet Research (IfZ), Göttingen, Germany; 2 Department Plant Viruses, Leibniz Institute DSMZ-German Collection of Microorganisms and Cell Cultures, Braunschweig, Germany; 3 Department Bioinformatics and Databases, Leibniz Institute DSMZ-German Collection of Microorganisms and Cell Cultures, Braunschweig, Germany; 4 Diagnostics Plant Diseases, Plant Protection Service, Chamber of Agricultur of Northrhine Westfalia, Diagnostics Plant Diseases, Plant Protection Service, Köln, Germany

**Keywords:** BCV1, *Capulavirus betae*, *Geminiviridae*, high-throughput sequencing, splicing, sugar beet

## Abstract

*Capulavirus betae* (beet capulavirus 1, BCV1) is a recently described species of the genus *Capulavirus* (family *Geminiviridae*) that was first identified in sugar beet in France. Capulaviruses are new group of geminiviruses because they are transmitted by aphids. Here, we report the identification and molecular characterization of an isolate of BCV1 infecting cultivated tomato in Germany and describe the construction of an infectious clone. In August 2025, tomato plants displaying severe virus-like symptoms, including leaf deformation, leaf curling, stunting and chlorosis were collected from two production sites in North Rhine-Westphalia. High-throughput RNA sequencing revealed a single viral contig representing a complete circular DNA genome with 99.5% nt identity to BCV1. The presence of the virus was additionally confirmed in five out of six individual tomato samples by specific PCR. Rolling-circle amplification (RCA) supported the circular nature of the genome and direct sequencing of RCA products confirmed the HTS-derived sequence. An infectious clone was generated and used to induce systemic infection after agrobacterium-mediated inoculation of sugar beet, tomato and *Nicotiana benthamiana*, thereby demonstrating that the cloned genome is biologically active in taxonomically distinct hosts. The RNA-seq data revealed a novel splicing event in the complementary-sense transcripts which was confirmed by RT-PCR. Together, these results expand the known host range and geographic context of BCV1 and establish an experimental system for studying a capulavirus pathosystem.

## Introduction

Geminiviruses are a large family of plant-infecting DNA viruses with small circular ssDNA genomes. Their genomes are either monopartite or bipartite and range from ~2.5 to 5.2 kb in total size and can be accompanied by satellites. A defining feature of the family is their characteristic geminate particle morphology, consisting of two incomplete icosahedral particles joined together and formed by a single coat protein. Geminiviruses replicate in the nuclei of infected plant cells via dsDNA intermediates, using a virus-encoded replication-associated protein and host replication factors to initiate rolling-circle replication and, in some cases, recombination-dependent replication [1, 2]. The family *Geminiviridae* comprises over 500 recognized species across 18 genera, including *Capulavirus*, *Begomovirus*, *Mastrevirus*, *Curtovirus* and others (https://ictv.global/files/proposals/approved). Among these, *Begomovirus* is by far the largest genus and includes many devastating crop pathogens. The current classification of geminiviruses reflects differences in genome organization, host range, insect vectors and phylogenetic relationships [2–4].

Geminiviruses are of major biological and agricultural importance. They infect both monocotyledonous and dicotyledonous plants and cause economically significant diseases in many crops, particularly in tropical and subtropical regions. Important species include *Begomovirus coheni* (tomato yellow leaf curl virus), *B. manihotis* (African cassava mosaic virus), *Begomovirus costai* (bean golden mosaic virus), *Curtovirus betae* (beet curly top virus, BCTV), *B. gossypimultanense* (cotton leaf curl Multan virus), *Mastrevirus storeyi* (maize streak virus) and *Mastrevirus hordei* (wheat dwarf virus). Typical symptoms include leaf curl, yellow mosaic, streaking, dwarfing, stunting and severe yield losses [1, 2]. A central feature of geminivirus biology is their close association with insect vectors. Different geminivirus genera are associated with distinct insect vector groups: begomoviruses are transmitted mainly by whiteflies of the *Bemisia tabaci* species complex; mastreviruses, curtoviruses, becurtoviruses and turncurtoviruses are transmitted by leafhoppers; grabloviruses and topocuviruses are transmitted by treehoppers; and capulaviruses are exceptional in that several members are transmitted by aphids [1, 2]. Experimentally demonstrated capulavirus vectors include *Aphis craccivora* for *Capulavirus medicagonis* (alfalfa leaf curl virus, ALCV) and *C. euphorbiae* (euphorbia caput-medusae latent virus, EcmLV) and *Dysaphis plantaginea* for *C. plantagonis* (plantago lanceolata latent virus, PILV); the vectors for the remaining capulavirus species, including *C. betae* (beet capulavirus 1, BCV1), *C. phaseoli* (French bean severe leaf curl virus, FbSLCV) and *C. trifolii* (trifolium virus 1, TrV1) are unknown [5–7].

Capulaviruses share the core features of the *Geminiviridae*, including circular ssDNA genomes, conserved replication-associated functions and geminate virions. However, they differ from most other geminiviruses due to their unusual genome organization and aphid transmission. They are monopartite geminiviruses with genomes of ~2.7 kb and a typical ambisense organization. Virion-sense ORFs are encoded on one strand, whereas complementary-sense replication-associated ORFs are encoded on the opposite strand. A distinctive feature of capulavirus genomes is their complex virion-sense region. Capulaviruses encode four virion-sense ORFs, V1, V2, V3 and V4. V1 is considered to encode the coat protein, whereas V3 and V4 contain predicted transmembrane features reminiscent of movement proteins of mastreviruses. Recent findings explicitly note that two or more of the V-side ORFs may form an intron-containing movement-protein-like gene and that V2 may be non-functional or may be expressed through a spliced transcript that fuses V2 and V4. On the complementary strand, capulaviruses encode C1, C2 and C3. It is inferred that C1 and C2 transcripts generate the functional Rep protein from a spliced transcript, while the unspliced C1 transcripts may encode a RepA-like protein. The C3 is embedded within C1 and is likely analogous to geminiviral C3/REn-like accessory proteins; however, the function(s) of capulavirus C3 remain uncharacterized [8, 9].

The development of high-throughput sequencing (HTS) approaches has clearly increased the number of identified novel geminiviruses. Together with the use of pairwise sequence comparisons, this has led to the rapid development of the taxonomy of these viruses [1, 3, 4]. BCV1 was recently identified through HTS in a pool of volunteer sugar beet plants collected in Normandy, France. Its complete genome comprises 2,744 nt and exhibits a capulavirus-like organization. The virus shares only limited genome-wide nt identity with previously known capulaviruses, falling below the accepted species demarcation threshold. Therefore, the name BCV1 was proposed for this novel virus; however, a natural isolate is lacking [10]. Currently, there is very limited experimental information available on symptomatology, infectious clone biology, insect vectors, mode of transmission, subcellular localization of viral proteins, transcript isoforms, the functions of each viral protein or molecular determinants of transmission. Here, we report the identification of a BCV1-related capulavirus in cultivated tomato in Germany and provide molecular evidence for its complete circular genome. We further describe the construction of an infectious clone and demonstrate its infectivity and symptom induction in sugar beet, tomato and *Nicotiana benthamiana*. Finally, we provide evidence of a novel splicing event in the complementary-sense transcripts. Together, these results expand the known biological context of BCV1, identify tomato as an experimental and naturally infected host and provide a foundation for future studies on capulaviruses.

## Methods

### Plant material and sample collection

In August 2025, tomato plants showing virus-like symptoms were sampled from two production sites belonging to organic farms in North Rhine-Westphalia, Germany. Symptoms included leaf deformation, leaf curling, stunting and chlorosis (Fig. 1). The plants were grown in greenhouses that were not insect-proof, which had a high density of weeds and potential insect vectors and were also propagated on-site from seed. Individual symptomatic plants were sampled for molecular diagnostics and HTS. Sugar beets, which can also apparently be infected, were not present in the surrounding area. Additional plant material used for infectivity assays included sugar beet (*Beta vulgaris*), tomato Moneymaker (*Solanum lycopersicum* cv. Moneymaker) and *N. benthamiana*. Plants were grown under controlled greenhouse conditions with day and night temperatures of 24 and 18 °C, respectively, and a 14 h photoperiod.

**Fig. 1.**
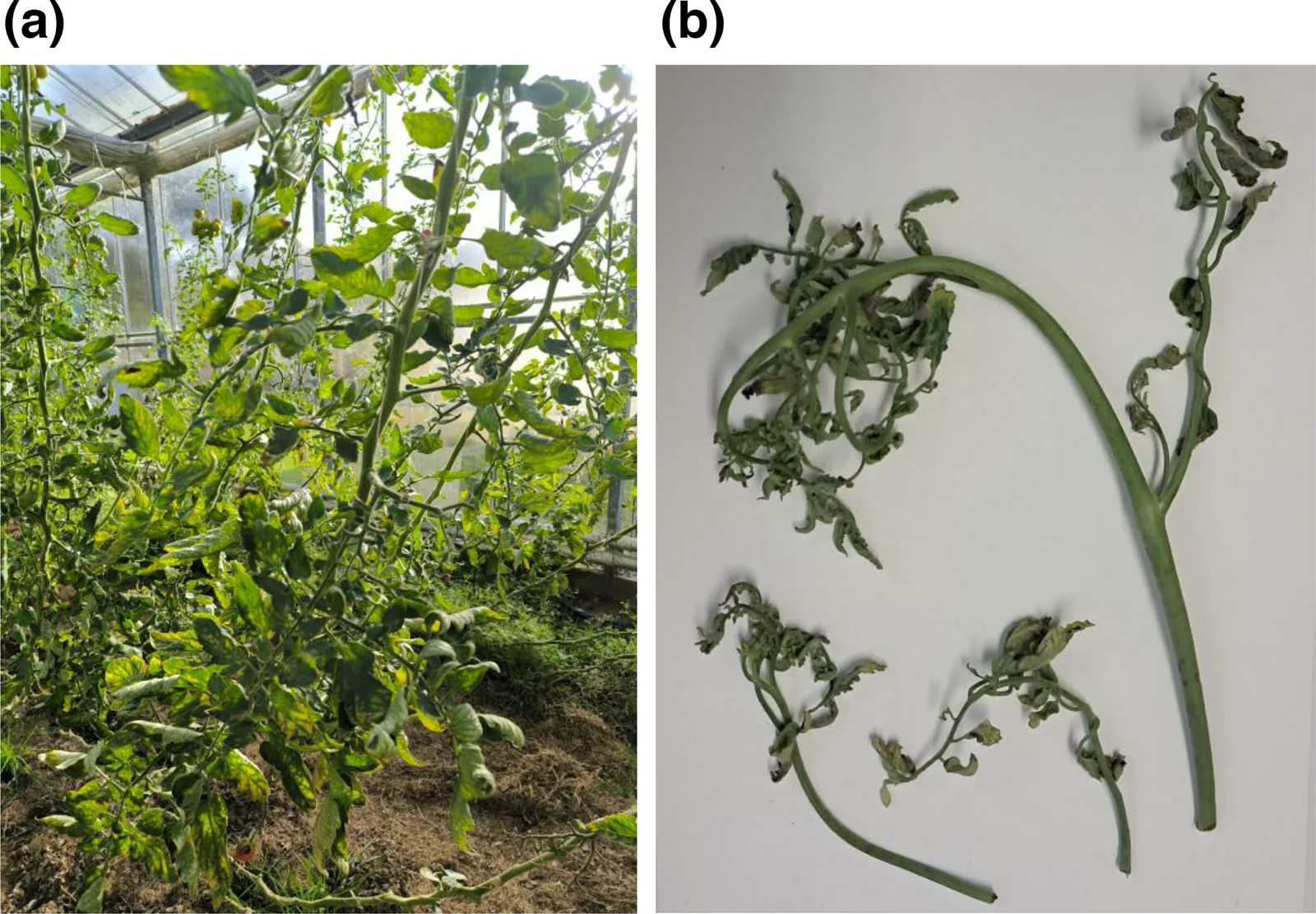
Symptoms in tomato plants. (**a**) The tomato plants showing symptoms of chlorosis and leaf curling, particularly on older leaves. (**b**) Severe leaf curling and stunting on younger shoots and leaves.

### Total RNA extraction and high-throughput sequencing

Total RNA was extracted from symptomatic leaves of two individual tomato plants using a Spectrum^®^ Plant Total RNA kit (Sigma), including an on-column DNase I treatment, according to the manufacturer’s instructions. A cDNA library was prepared from a pooled sample of the two RNAs according to the NEBNext UltraExpress RNA Library Prep kit (New England Biolabs), including a ribosomal RNA depletion treatment (QIAseq FastSelect, Qiagen), and sequenced on an Illumina NextSeq 2000 platform, with a paired-end read (2×150 bp) strategy. The bioinformatic analysis was conducted in Geneious Prime version 2026.0.2 (Dotmatics, USA) according to a custom workflow developed in-house at Plant Virus Department, DSMZ. In brief, the raw reads were paired, quality-trimmed, normalized, merged and aligned to host sequences, including chromosome sequences, predicted transcripts (BioProject PRJNA66163), chloroplast sequence (NC_007898) and mitochondrial sequence (NC_035963), using the Geneious mapper (20% maximum mismatches per read). Unmapped reads were *de novo* assembled with the Geneious assembler and the obtained contigs were screened by blastn and against a custom reference database for virus discovery and taxonomic assignment.

### DNA extraction, PCR and rolling-circle amplification

DNA was extracted from six individual tomato samples and used for BCV1-specific PCR and rolling-circle amplification (RCA).

The extracted DNA was tested for the presence of BCV1 using the cycling conditions and the primers Capu-RepF and Capu-RepR, which amplify a 531 bp fragment of the replicase, according to Zhang *et al*. [10]. The identity of the amplicons was verified by direct forward and reverse Sanger sequencing (Microsynth Seqlab, Germany). In addition, circular DNA was amplified using phi29 polymerase and the TempliPhi Kit (Merck, Darmstadt, Germany) according to the manufacturer’s protocol. A total of ~20 ng of total nucleic acids was dissolved in 5 µl of sample buffer, denatured at 95 °C for 3 min and cooled down to room temperature. After adding 5 µl reaction buffer and 0.2 µl of the enzyme mix, the reaction was incubated for 18 h at 30 °C and then stopped at 65 °C for 10 min. RCA products were analysed by gel electrophoresis and selected products were subjected to either direct sequencing or amplification of virus-specific fragments.

### Amplification of the full-length genome and sequence analysis

Using back-to-back primer pair BC-EcoR F/R (Table S1, available in the online Supplementary Material), the full-length BCV1 genome (ca. 2.8 kb in size) was amplified in DNA extracts from two tomato samples using PCR master mix with Phusion high-fidelity DNA polymerase (Thermo Fisher Scientific). PCR products were cloned into a pJet1.2 vector (Thermo Fisher Scientific, USA) and sequenced using Sanger plasmid sequencing service (Microsynth Seqlab, Germany). To identify similarity between the sequences and the publicly available sequences, the sequences were blast searched at NCBI (https://blast.ncbi.nlm.nih.gov/Blast). To demonstrate the similarities and relationship between full-length and individual ORFs of the amplified virus and other members of capulaviruses, phylogenetic trees were constructed using the maximum likelihood method and the Jones-Taylor-Thornton (JTT) model. Evolutionary analyses were conducted in mega X [11].

### Construction of an infectious clone of BCV1 and plant inoculation

A 0.3-mer DNA fragment of the viral genome spanning the large intergenic region (0.9 kb in size) was amplified by PCR using the primer pair BC-Eco-F/BC-Bam-R (Table S1) from infected tomato plant DNA extracts. This DNA fragment was cloned into the pLHAB binary vector (GenBank accession number AY234325) at the *Eco*RI/*Bam*HI sites to produce pLHAB-BCV 0.3 mer construct. The full-length DNA fragment was then sub-cloned into the *Eco*RI restriction enzyme site of the pLHAB-BCV 0.3 mer to produce an infectious clone, pLHAB-BCV 1.3 mer (Fig. 2). Two of the produced clones were sequenced and transferred into *Agrobacterium* cells (C58C1) by electroporation (Multiporator, Eppendorf). *Agrobacterium* cells containing infectious clones BTV1 were grown in LB medium until they reached an OD=0.5 and used for the inoculation of tomato, *N. benthamiana* and sugar beet seedlings. To test systemic infection, 12 plants from each species were inoculated at hypocotyls at the three-leaf stage using micro-fine U-100 insulin syringe. For the negative control, *Agrobacterium* cells containing the empty pLHAB vector were injected into six plants of each species. The plants were monitored for the production of viral symptoms starting at 10 days after inoculation.

**Fig. 2.**
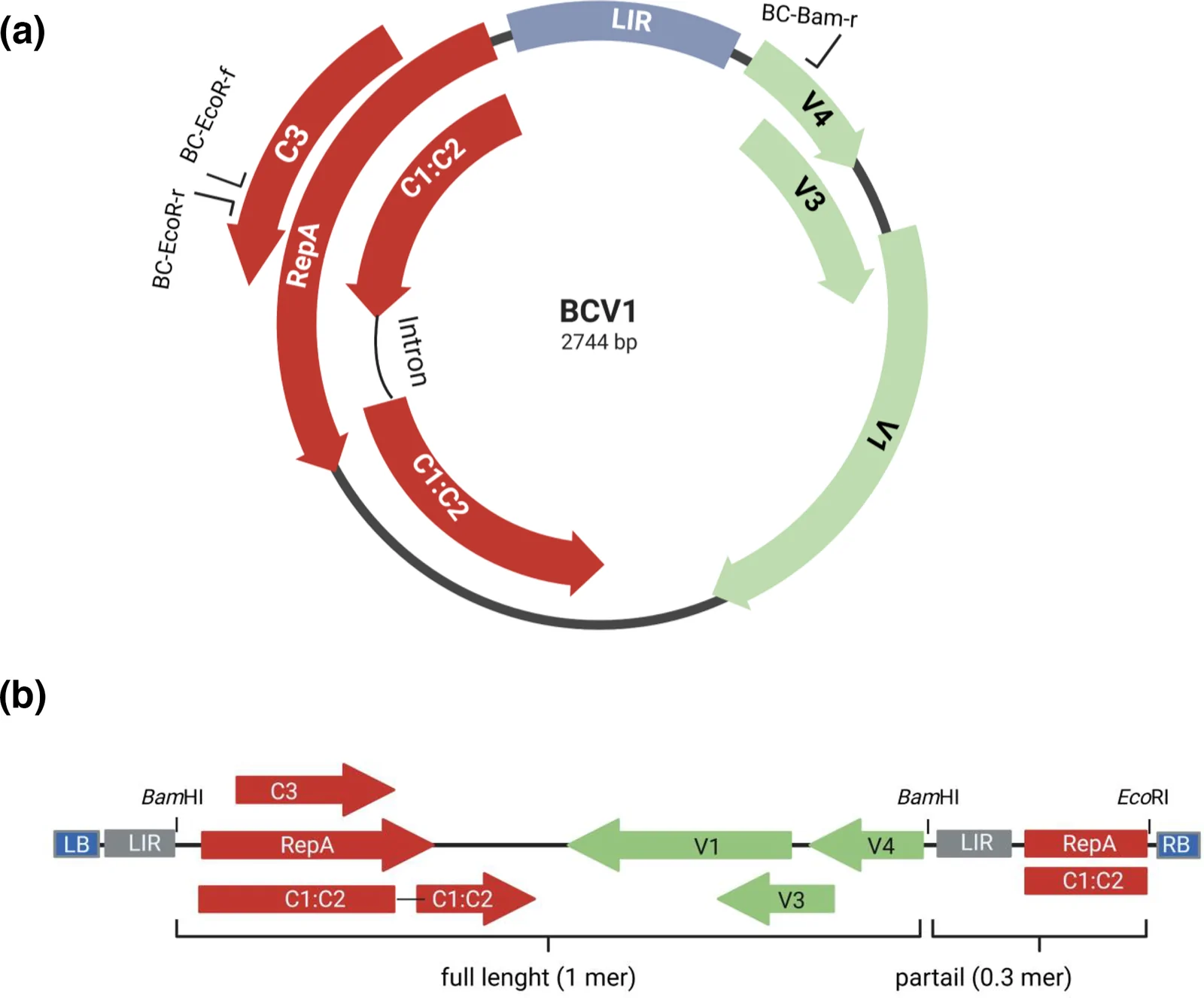
Schematic maps of the BCV1 genome and the infectious clone. (**a**) The full-length BCV1 genome shows the positions of ORFs and the designed primers used to amplify the full and partial genomes. (**b**) The genomic map of the infectious clone and the positions of the restriction enzymes used to subclone the 1.3-mer viral genome between the T-DNA borders of the binary vector pLHAB are shown.

### Quantification of viral DNA in plants

Total DNA was extracted from the leaf tissues with curly symptoms using KingFisher Duo Prime instrument (Thermo Fisher Scientific). As a control, DNA was extracted from leaf tissues of healthy plants. To perform the quantitative PCR (qPCR), 100 ng of extracted DNA was used in a reaction volume of 15 µl containing 1× iTaq Universal SYBR Supermix (Bio-Rad) and 0.330 µM of each primer (CA-F and CA-R; Table S1). The qPCR was carried out in a CFX96 Real-Time System C1000 Touch Thermal Cycler (Bio-Rad, Germany). The reaction conditions were as follows: an initial denaturation at 95 °C for 3 min followed by 40 cycles of 95 °C for 30 s and 60 °C for 20 s. Melting curve analysis (60–95 °C) was performed after amplification to check the specificity of the reaction. Each biological sample was analysed in three technical replicates. Data normalization and calculation of relative expression values were done using the 2^−ΔCt^ method [12]. The sugar beet glyceraldehyde-3-phosphate dehydrogenase gene (KJ784472), the *N. benthamiana* F-box (XM_009790698) and the tomato elongation factor 1-α-like gene (NM_001279325) were used as endogenous controls to normalize viral DNA replication in each infected plant. Six biological replicates were tested for each virus and statistically analysed using R software (Tukey test, *P*<0.05).

### Analysis of viral transcript splicing

To identify putative viral splice variants, RNA-seq reads obtained from infected tomato tissue were quality-filtered and adapter-trimmed using fastp v0.23.4 [13]. Trimmed reads were subsequently mapped to the viral genome sequence using the STAR aligner v2.7.11b in splice-aware mode [14]. STAR-reported splice junctions were extracted and evaluated according to the genomic coordinates of the predicted intron, strand orientation, splice-junction motif, number of uniquely and multi-mapped reads spanning the junction and maximum spliced alignment overhang. Candidate viral splice junctions were then manually inspected in the Integrative Genomics Viewer v2.16.1 to assess read support, junction consistency and mapping plausibility. For comparative screening of viral RNA-seq datasets, prospective splice junctions were prioritized when supported by at least five junction-spanning uniquely mapped reads or at least five junction-spanning multi-mapped reads. In the BCV1 dataset, where the total number of uniquely mapped viral read pairs was low, STAR-predicted junctions were also retained for manual inspection when supported by fewer reads. Since the initial analysis of the sequencing data resulted in very few reads per splice site, a second RNA-seq dataset with more reads was obtained from a mixture of three agroinfected tomato plants. This dataset was analysed in the same way as the first one. This provided that the splice junctions were reproducible and biologically plausible in the context of the viral genome organization.

### RT-PCR and sequencing the spliced transcripts on the complementary-sense genes

Leaf tissues were collected from healthy sugar beet, *N. benthamiana* and tomato plants, as well as from BCV1-infected plants displaying leaf curling symptoms. Total RNA was extracted using a Direct-zol RNA Miniprep kit (Zymo Research, Freiburg, Germany). The quantity and purity of the RNA (OD260/280≥2.0) were determined using photometry (NanoDrop) and agarose gel electrophoresis. For reverse-transcription PCR (RT-PCR), 1 µg of DNase I‐treated RNA from BCV1-infected and control plants was used for cDNA synthesis using reverse transcriptase (Thermo Fisher Scientific, USA) and random hexamer primers. RT-PCR reactions were carried out in a final volume of 20 µl, consisting of 10 µl of ‘Phusion Flash’ PCR Master Mix (2×) (Thermo Fisher Scientific, Waltham, MA, USA), 0.5 µM of each primer spanning the identified splicing regions and 2 µl of template cDNA. The CA-Rep-F/CA-R primers span the C1:C2 splicing region and are expected to amplify two cDNA fragments with 289 and 438 nt in size. The CA-F/CA-Rep-R primers span a new C1 splicing region and are expected to amplify two cDNA fragments with 429 and 532 nt in size. The PCR conditions were 98 °C for 5 s, 30 cycles at 98 °C for 10 s, 50 °C for 5 s, 72 °C for 5 s, followed by a final step at 72 °C for 2 min. In all RT-PCR assays, 100 ng of RNA from infected plants was used to control for possible DNA contamination.

## Results

### High-throughput RNA sequencing and RCA reveals BCV1 infection in symptomatic tomato plants

Tomato plants from two production sites in North Rhine-Westphalia, Germany, showed severe virus-like symptoms, including leaf curling and plant deformation. Initial diagnostic for the presence of tobamoviruses and Pepino mosaic virus failed to identify common tomato infecting viruses (data not shown). To identify the possible causal agent, high-throughput RNA sequencing was performed. The Illumina sequencing generated 5,976,308 raw reads, of which 5,831,338 reads remained after quality trimming and were used for *de novo* assembly. A total of 7,809 contigs were generated. blastn searches identified only one contig, contig 13, as a plant virus-derived sequence. Further sequence curation and extension resulted in the reconstruction of a circular viral genome. The assembled sequence showed highest nt identity to BCV1 (GenBank accession PQ682516), recently described from sugar beet in France, with 99.5% nt identity and 100% query coverage. No additional plant virus-like sequences were detected in the contig dataset, identifying BCV1 as the unique candidate viral agent associated with the symptomatic tomato plants.

BCV1-specific PCR confirmed the presence of the virus in five of six individual tomato samples. RCA was positive in the same samples, supporting the presence of circular viral DNA. These results demonstrated that symptomatic tomato plants from Germany were infected with a circular DNA virus closely related to BCV1.

### RCA-based cloning and sequence validation of the tomato-derived BCV1 genome

To obtain an infectious clone, RCA product from BCV1-positive tomato plants were used as template for PCR amplification with back-to-back primers designed from the RNA sequencing-derived genome sequence. The complete genome sequence of 2,744 nt, containing the expected nonanucleotide motif ‘TAATATTAC’, has been deposited in GenBank under accession number PZ490328, providing a novel sequence entry for BCV1 in addition to the only one available so far. Thus, a 1.3 mer of BCV1 genome (Fig. 2b) was constructed for agrobacterium-mediated inoculation. The RCA-derived cloned genome was sequenced again to confirm the identity and integrity of the viral genome. The validated clone sequence was used for subsequent genome annotation and phylogenetic analyses.

### Genome organization and phylogenetic relationship of the tomato-derived BCV1 isolate

The validated tomato-derived BCV1 genome was 2,744 nt in length and showed the typical genome organization of capulaviruses. The predicted virion-sense ORFs included the coat protein gene V1 and the upstream V3 and V4 regions. The complementary-sense ORFs included C1, C2 and C3, consistent with the capulavirus genome model in which C1 and C2 contribute to the functional Rep protein through splicing (Fig. 2a).

Phylogenetic analysis based on complete genome sequences placed the tomato-derived BCV1 isolate (GenBank accession number PZ490328) together with the BCV1 isolate previously identified from sugar beet. Both isolates formed a well-supported clade that was clearly separated from other known capulavirus species. PILV isolates represented the closest relatives outside the BCV1 clade. Pairwise sequence comparisons showed that the tomato-derived BCV1 isolate shared only 50.2–60.8% nt identity with other capulavirus members, supporting its assignment to BCV1 while clearly distinguishing it from other capulavirus species (Fig. 3a). Additional phylogenetic analyses based on individual virion-sense and complementary-sense genes, including V1, V3, V4, C1, C1:C2 and RepA, confirmed that the tomato and sugar beet BCV1 isolates cluster together and are distinct from other capulaviruses. These analyses support the identification of the tomato-associated virus as a BCV1 isolate (Fig. 3b).

**Fig. 3.**
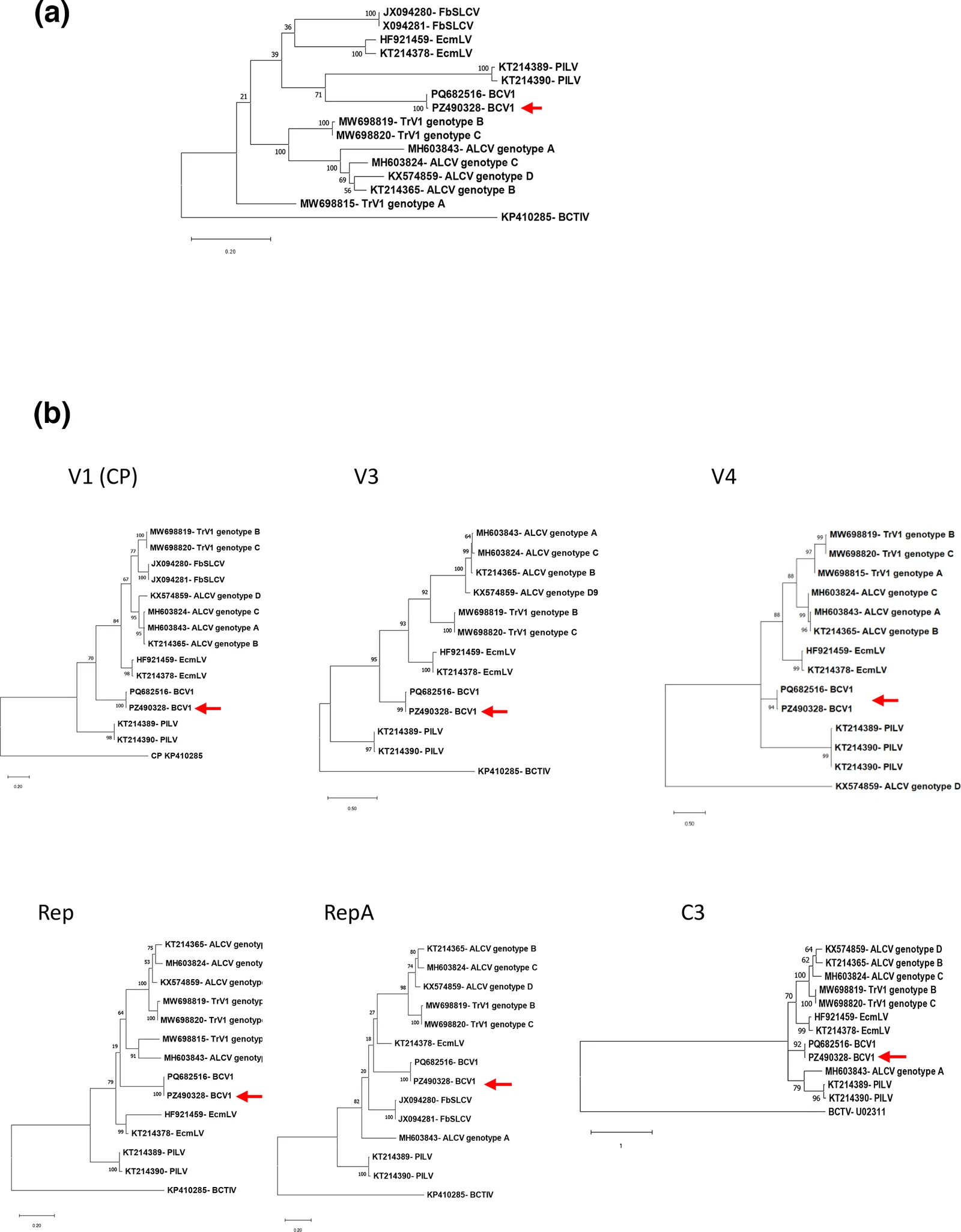
Phylogenetic analysis of the complete genome (**a**) and gene sequences (**b**) of the BCV1 tomato isolate and representative members of reported capulaviruses. Phylogenetic trees were constructed using the maximum likelihood method and the JTT model. Evolutionary analyses were conducted in MEGA X. Numbers on branches are bootstrap values based on 1,000 replicates. Representative genome sequences of capulaviruses including ALCV, EcmLV, PILV, FbSLCV and TrV1 were used in the analysis. The BCTIV genome sequence was used as the outgroup. The scale bar represents 2 nt substitutions per 100 nts. The BCV1 sequence in this study is indicated by an arrow.

### Infectivity of the BCV1 1.3-mer clone in tomato, sugar beet and *N. benthamiana*

The infectious 1.3-mer BCV1 clone was used for agrobacterium-mediated inoculation of tomato, sugar beet and *N. benthamiana* (Fig. 4a). BCV1 infection was confirmed in all three plant species, demonstrating that the cloned tomato-derived BCV1 genome is infectious. In *N. benthamiana*, leaf curling developed on young systemic leaves at ~12 days post inoculation (dpi). In tomato, leaf curling and deformation were observed at ~18 dpi. In sugar beet, symptoms developed later and were generally milder, with leaf curling and vein swelling appearing at ~28 dpio. These results show that the tomato-derived BCV1 isolate can systemically infect tomato, sugar beet and *N. benthamiana*, with symptom development and timing differing between host species. BCV1 DNA accumulation was quantified in infected tomato, sugar beet and *N. benthamiana* plants by real-time PCR (Fig. 4b). Viral DNA was detected in all three host species. However, BCV1 DNA levels were higher in tomato and *N. benthamiana* than in sugar beet. This pattern was consistent with the more pronounced and earlier symptom development observed in tomato and *N. benthamiana*, whereas sugar beet showed delayed and milder symptoms.

**Fig. 4.**
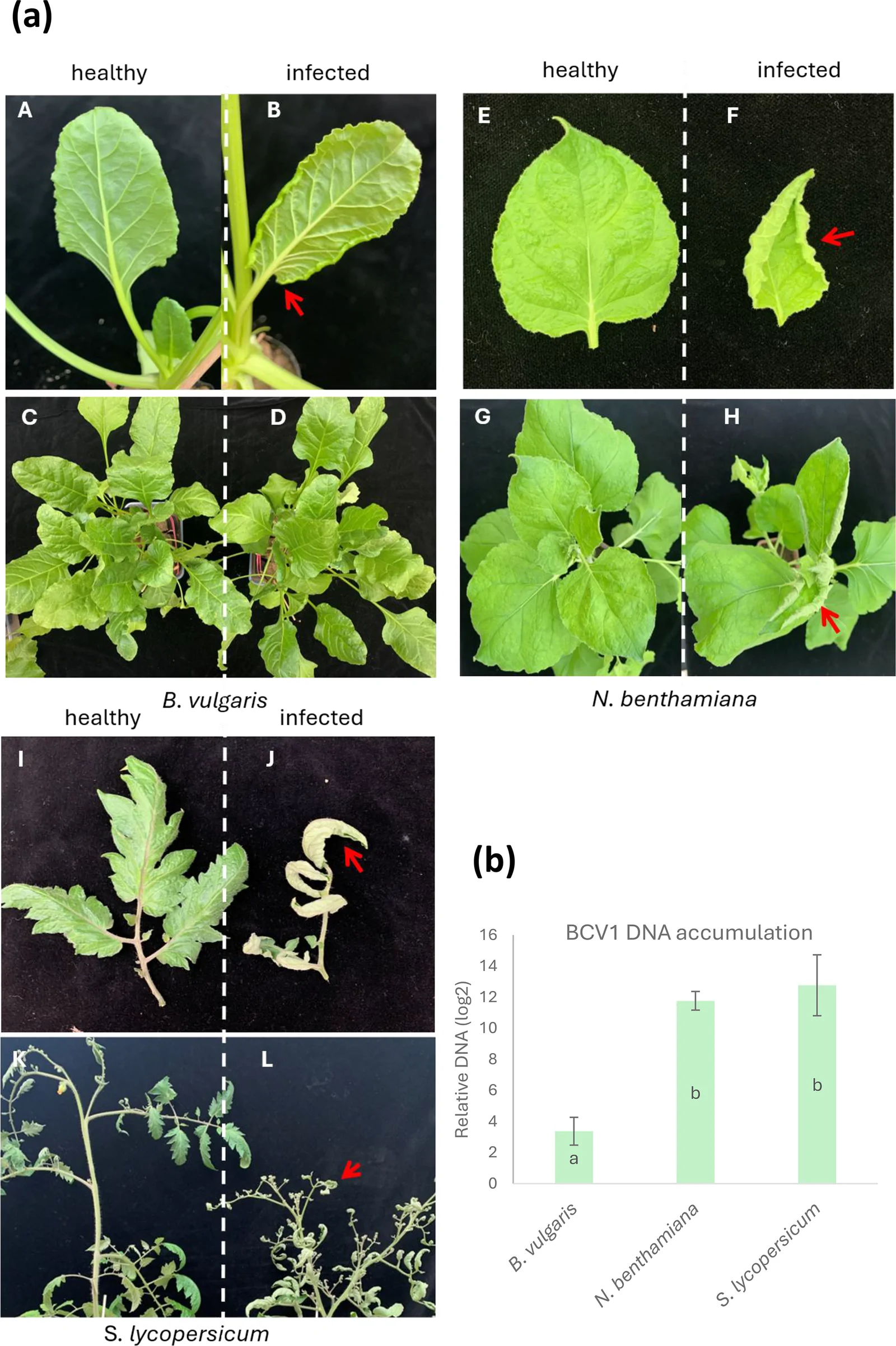
Symptom development and viral DNA quantification in BCV1-infected plants. (**a**) Panels A, C, E, G, I and K are healthy controls. Read arrows indicate the mild leaf curling and vein swelling in young leaves in sugar beet plants (B and D); severe upward leaf curling in *N. benthamiana* (F and H) and tomato (J); the infected tomato plants had small leaves (I). Symptoms appeared in *N. benthamiana* and tomato after 12 and 21 days post inoculation (dpi). In sugar beet symptoms appeared after 28 dpi. (**b**) Real-time PCR shows the viral DNA accumulation in BCV1-infected plants. The error bars show the sd values for six biological replicates. Bars with the same letter indicate statistically insignificant (*P*<0.05).

### RNA-seq supports complementary-sense splicing in BCV1

Assessment of the viral RNA-seq data revealed splice junctions on complementary-sense transcripts. One junction was consistent with the expected C1:C2 splicing event predicted to generate the functional Rep protein. An additional splice junction was detected within the C1 region and is predicted to generate a C1:C3 fusion protein containing a short C1-derived N-terminal segment fused to the C3 ORF. In the first assay, based on RNA seq data from the naturally infected tomato plants, a total of 4,665 mapped reads to BCV1 genome. In this dataset, one and three reads were mapped to the of C1:C2 and the novel C1 intron junctions, respectively. In the second assay, based on a total of 10,056 mapped reads to BCV1 genome, 7 and 17 reads were mapped to the intron junction of C1:C2 and the novel C1 intron, respectively. These findings suggest that transcript splicing may expand BCV1 coding potential.

RT-PCR and Sanger sequencing of splice junctions confirmed the presence of intron (intron I; 139 nt in size) in C1:C2 transcript and an additional intron (intron II; 103 nt in size) in the C1:C3 transcripts (Fig. 5a). These RT-PCR sequences validate the candidate transcript isoforms (Fig. 5b) identified in the RNA-seq data. The splice-junction coordinates and canonical splice-site orientation are consistent with splicing of complementary-sense C1 transcripts. Furthermore, although the RNA-seq libraries were non-strand-specific, the reads were only mapped to C1 transcripts in the complementary sense. No reads were detected for the spliced regions on the virion sense C1 transcripts.

**Fig. 5.**
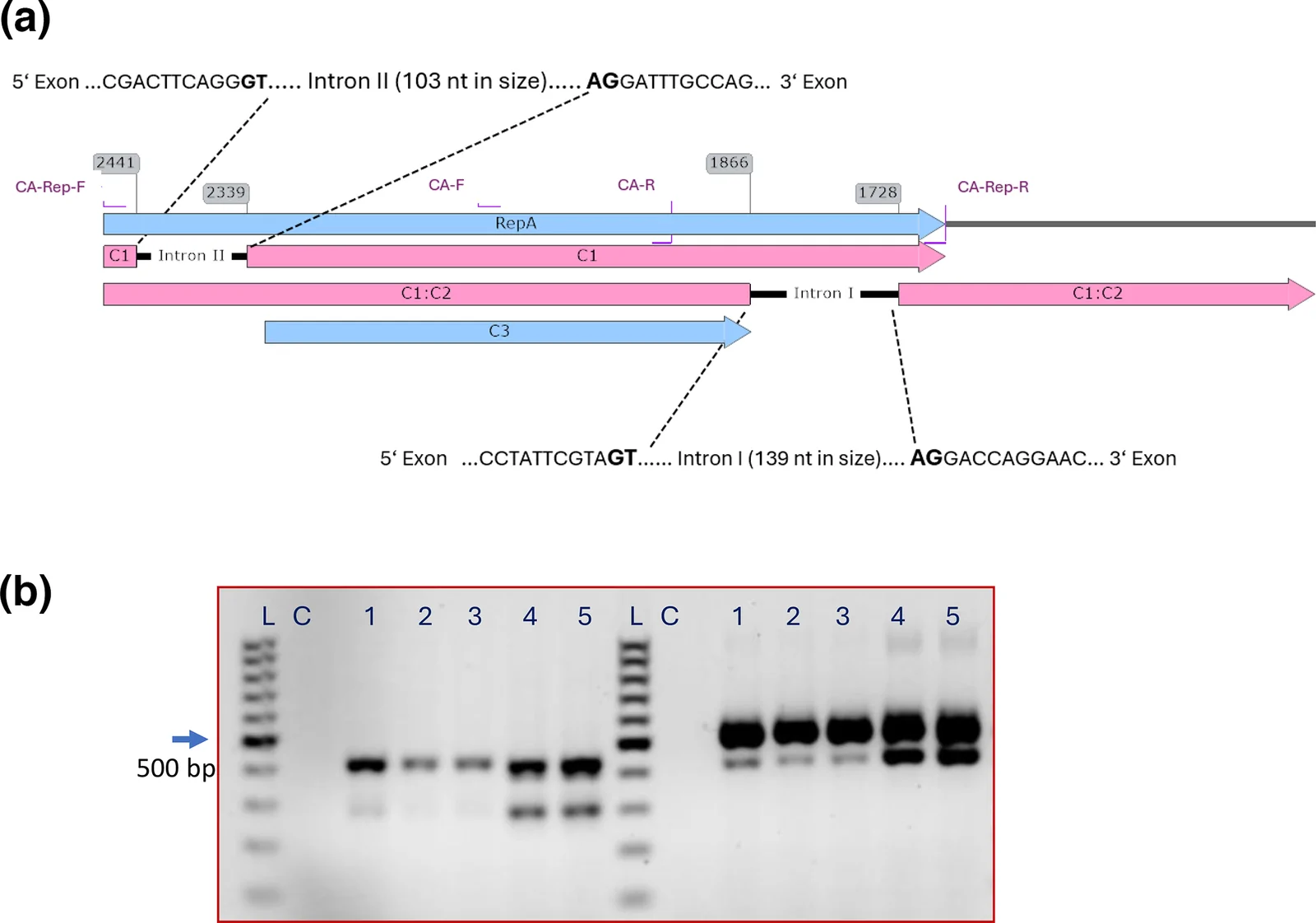
The schematic map shows the complementary sense transcripts of BCV1. (**a**) The splicing donor and acceptor sites on the C1 transcripts containing either intron I or intron II are indicated in bold. The positions of the primers used in RT-PCR are shown. (**b**) The RT-PCR assay shows amplification of the BCV1 C1 and C2 transcripts in infected plants. Three representative sugar beet plants (lanes 1–3), one tomato (lane 4) and one *N. benthamiana* plant (lane 5) were tested in the RT-PCR assay. The left side gel shows amplification of cDNA using CA-F/CA-Rep-R primers spanning the C1:C2 splicing region. The gel picture on the right side shows cDNA amplification using CA-Rep-F/CA-R primers spanning the novel C1 splicing region. An RNA sample was used as a control. A 100 bp DNA ladder was used.

## Discussion

BCV1 has been reported as a new capulavirus that infects sugar beet in France [10]. However, due to co-infection with the beet yellows virus, the pathogenicity and symptom development in sugar beet are unknown and an isolate was not obtained. In addition, data mining of a publicly available *Thalictrum thalictroides* whole-genome shotgun assembly from the USA identified a 3,125-nt contig representing a longer-than-unit-length, likely episomal BCV1-related sequence. This sequence corresponds to a 2,744-nt unit-length genome and shares 99.4% nt sequence identity with the French beet isolate, while symptoms in *T. thalictroides* are not known [10]. Our study reports the first identification and molecular characterization of a BCV1-related capulavirus infecting cultivated tomato in Germany. The detection of BCV1 in tomato substantially expands the biological context of this newly described virus. The present finding in tomato, together with the successful infection of tomato, sugar beet and *N. benthamiana* using an infectious clone, indicates that BCV1 can infect taxonomically distinct hosts. Additionally, the BCV1 genome alone is sufficient to establish infection in experimental host plants and provide a platform for reverse genetics. This is particularly important for capulaviruses because the functions of putative V2, V3 and V4 remain largely unknown. The availability of an infectious clone now enables the targeted mutagenesis of these ORFs, the analysis of putative splice sites and the comparison of replication, movement, symptom development and vector transmission.

Quantification of viral DNA revealed a low level of the virus in sugar beet plants compared to tomato and *N. benthamiana* under controlled greenhouse conditions. This may explain the development of severe leaf curling in tomato and *N. benthamiana* compared to the mild leaf curling in the infected sugar beet plants. Supporting this result, symptom development and severity were also found to be positively correlated with viral DNA accumulation in *Becurtovirus betae* (beet curly top Iran virus, BCTIV)-infected tomato plants [15] and in the Arabidopsis plants infected with BCTV [16]. Various responses of host plants to other members of capulaviruses have been reported. For example, ALCV produced clear leaf curling symptoms in alfalfa [9], whereas EcmLV, TrV1 and PILV remained asymptomatic in *Euphorbia caput-medusae*, *Trifolium repens* (white clover) and *Plantago lanceolata*, respectively [6, 9, 17]. This indicates that the development of symptoms by members of capulaviruses including BCV1 depends on the interaction of each virus with host factors. These viruses lack the C4 ORF, which is responsible for symptom production in members of geminiviruses [18]. The gene responsible for pathogenicity and symptom production in capulaviruses is so far unknown.

Phylogenetic analysis of the complete BCV1 genome sequence showed that the tomato isolate and sugar beet isolates clustered together. The PILV isolates were the closest capulavirus to the BCV1 isolates. This is in line with the genome organization in BCV1 and PILV as both lack V2 ORF [6, 10]. Pairwise sequence comparisons of the BCV1 genome showed 99.4% sequence identity between the tomato and the sugar beet isolates. The sequence identity between these isolates and other members of capulaviruses was in the range of 50.2–60.8% which is below the proposed 78% identity threshold as the potential species cutoff value for the genus designation in geminiviruses [4]. These results suggest that the tomato and sugar beet isolates should be regarded as belonging to a single BCV1 species.

RNA splicing and the presence of introns have been reported in several geminiviruses. In addition to capulaviruses, RepA and Rep proteins are either inferred or proven to be expressed from alternatively spliced complementary-sense transcripts in grabloviruses, becurtoviruses, mastreviruses and mulberry mosaic dwarf-associated virus [4, 19–21]. Furthermore, an intron has been identified in the movement protein of mastreviruses [20], and an intron has been inferred to occur in the movement protein of EcmLV [9]. RNA sequencing and RT-PCR assays for the complementary-sense transcripts in BCV1-infected plants indicated a splicing event in the C1:C2 region, which is consistent with the genome organization in capulaviruses. The identified C1:C2 splice event likely generates the functional Rep protein, as proposed for other capulaviruses and related geminiviruses [4, 9]. Identification and sequence characterization of an additional unique splicing event in the C1 transcripts suggest that BCV1 may produce alternative transcript isoforms, although their biological relevance remains to be determined. Future strand-specific RNA-seq, long-read transcript analysis and proteomics will be important to define the complete viral transcripts and protein expression patterns.

A key unresolved question is how BCV1 is transmitted in nature. Several capulaviruses are aphid-transmitted, and ALCV transmission by *A. craccivora* is highly specific and circulative. Given the broad natural host range currently associated with BCV1, spanning sugar beet, tomato, Arabidopsis and several wild herbaceous hosts, a highly polyphagous aphid such as *Myzus persicae* and *Aphis craccivora* represents the most plausible vector candidate. However, aphid transmission of BCV1 remains to be experimentally demonstrated. The detection of BCV1 in tomato production, the negative seedling tests from the original seed batches and the known biology of related capulaviruses suggest that aphid transmission is a plausible route that requires experimental testing. Identifying the vector species and mode of transmission is essential for understanding the epidemiology and risk potential of BCV1.

## Supplementary material

10.1099/jgv.0.002347 Table S1.

## Abbreviations

ALCV: alfalfa leaf curl virus
BCTIV: beet curly top Iran virus
BCTV: beet curly top virus
BCV 1: beet capulavirus 1
EcmLV: euphorbia caput-medusae latent virus
FbSLCV: French bean severe leaf curl virus
HTS: high-throughput sequencing
PILV: plantago lanceolata latent virus
qPCR: quantitative polymerase chain reaction
RCA: rolling-circle amplification
TrV1: trifolium virus 1
