# Supplementary material for: Molecular characterization and infectious clone construction of beet capulavirus 1 infecting tomato in Germany
Source: J Gen Virol. 2026 Oct 8;107(10):002347. doi: 10.1099/jgv.0.002347 (PMC13649057; doi:10.1099/jgv.0.002347)
Supplement: Supplementary file 1 [file jgv-107-02347-s001.pdf]

Table S1. list of primers used in this study

Primer name	Sequence (5'- 3')	Accession number
FBOX-Nb-F	GGCACTCACAAACGTCTATTTC	XM_009790698
FBOX-Nb-R	ACCTGGGAGGCATCCTGCTTAT	
SlEFα1-F	TACTGGTGGTTTTGAAGCTG	X14449.1
SlEFα1-R	AACTTCCTTCACGATTTCATCATA	
GAPDH R	GGA TCT CCT CTG GGT TCC TG	KJ784472
GAPD-F	TGGTGATAGTGGGCTGTAAAGG	
BC-EcoR-F	GAGGAATTCGGACTTGGATGTGG	PZ490328
BC-EcoR-R	TCCGAATTCCTCGCCAGATGTGTTC	
BC-Bam-R	CGTGGATCCATCAATATAAATT	
CA-F	ACACAGTAATGGGCTCTGG	
CA-R	GCAAGTAGAAGAAGCCCTAAG	
CA-Rep-F	ATGCCTAGACAAAGTCGTTTC	
CA-Rep-R	TCAATTATTAATGTGACAGAC	
